# Histologically confirmed isolated IgG4-related hypophysitis: two case reports in young women

**DOI:** 10.1530/EDM-14-0062

**Published:** 2014-09-01

**Authors:** Gabriela Alejandra Sosa, Soledad Bell, Silvia Beatriz Christiansen, Marcelo Pietrani, Mariela Glerean, Monica Loto, Soledad Lovazzano, Antonio Carrizo, Pablo Ajler, Patricia Fainstein Day

**Affiliations:** 1 Department of Endocrinology, Metabolism and Nuclear Medicine, Hospital Italiano, Perón 41901202, Buenos Aires, Argentina; 2 Department of Pathology, Hospital Italiano, Perón 41901202, Buenos Aires, Argentina; 3 Department of Radiology, Hospital Italiano, Perón 41901202, Buenos Aires, Argentina; 4 Department of Neurosurgery, Hospital Italiano, Perón 41901202, Buenos Aires, Argentina

## Abstract

IgG4-related hypophysitis is a recently described entity belonging to the group of IgG4-related diseases. Many other organs can also be affected, and it is more common in older men. To date, 32 cases of IgG4-related hypophysitis have been reported in the literature, 11 of which included confirmatory tissue biopsy and the majority affecting multiple organs. The aim of this report is to present two cases of biopsy-proven IgG4-related hypophysitis occurring in two young female patients with no evidence of involvement of other organs at the time of diagnosis.

**Learning points:**

- IgG4-related hypophysitis belongs to the group of IgG4-related diseases, and is a fibro-inflammatory condition characterized by dense lymphoplasmacytic infiltrates rich in IgG4-positive plasma cells and storiform fibrosis.
- It is more common in older men, but young women may also present this type of hypophysitis.
- Although involvement of other organs is frequent, isolated pituitary disease is possible.
- Frequent clinical manifestations include anterior hypopituitarism and/or diabetes insipidus.
- The diagnosis may be confirmed with any of the following criteria: a pituitary biopsy with lymphoplasmacytic infiltrates, with more than ten IgG4-positive cells; a sellar mass and/or thickened pituitary stalk and a biopsy-proven involvement of another organ; a sellar mass and/or thickened pituitary stalk and IgG4 serum levels >140 mg/dl and sellar mass reduction and symptom improvement after corticosteroid treatment.
- Glucocorticoids are recommended as first-line therapy.

## Background

Hypophysitis is a rare entity characterised by chronic inflammation of the pituitary, and is classified as primary and secondary based on its aetiology. Secondary hypophysitis includes cases resulting from other sellar lesions (Rathke cleft cyst, craniopharyngioma, germinoma, and pituitary adenomas); from therapeutic use of immunomodulatory drugs (CTLA4 blocking antibody, interferon α) or as a consequence of systemic diseases such as Wegener's granulomatosis, tuberculosis, sarcoidosis or syphilis (1).

The primary forms comprise five histopathological subtypes: lymphocytic or autoimmune, granulomatous, xanthomatous, necrotising, and IgG4-positive plasma cell-rich hypophysitis (1). Lymphocytic hypophysitis is the most common subtype, showing extensive pituitary infiltration by lymphocytes, predominantly affecting women between 30 and 40 years of age, often occurring during pregnancy or in the *postpartum* period, and frequently associated with other autoimmune disorders (2). IgG4-related hypophisitis is a recently described entity belonging to the group of IgG4-related diseases first described in 2001 by Hamano *et al*. (3) in a patient with sclerosing pancreatitis. We now know that many other organs can also be affected and that it is more common in older men (4).

To date, 32 cases of IgG4-related hypophysitis have been reported in the literature, 11 of which included confirmatory tissue biopsy (5) and the majority affecting multiple organs. The aim of this report is to present two new cases of biopsy-proven IgG4-related hypophysitis occurring in two young female patients, with no evidence of involvement of other organs at the time of diagnosis.

## Case reports

### Case 1

#### Case presentation

A 25-year-old woman presented to our clinic with a history of secondary amenorrhoea for the past eight months. Her relevant past history revealed chronic anaemia due to thalassaemia minor, menarche at age 13 years and no prior pregnancies. She was found to present normal vital signs and her physical examination was unremarkable. She was not taking any prescribed medication.

#### Investigation

**Figure 1:**
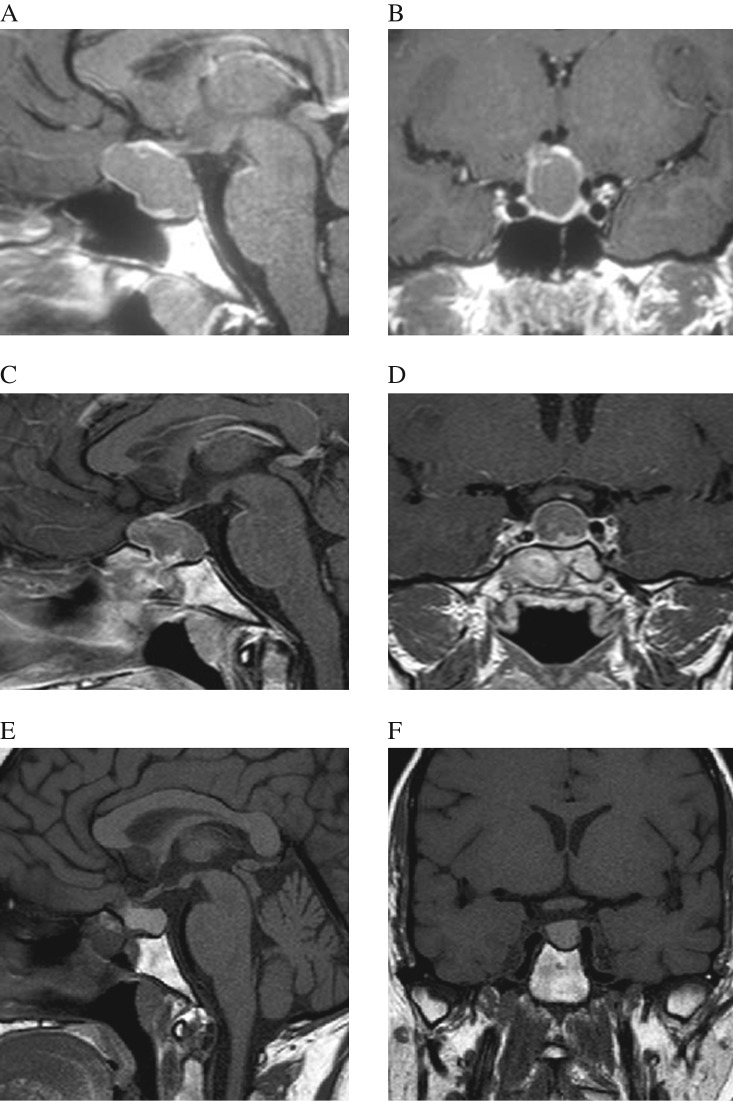
(A and B) Preoperative gadolinium-enhanced T1 weighted MRI: lesion measuring 19 mm vertical length near the optic chiasm, with irregular peripheral enhancement. (C and D) Postoperative gadolinium-enhanced T1 weighted MRI: persistence of heterogeneous asymmetrical mass with infiltration of the sphenoidal sinus near the optic chiasm. (E and F) Nonenhanced MRI: significant reduction in tumor size 4 months after starting meprednisone treatment.

Initial laboratory tests revealed hypogonadotrophic hypogonadism without hyperprolactinaemia and possible but not proven secondary adrenal failure (Table 1). Brain magnetic resonance imaging (MRI) revealed a sellar tumour with suprasellar extension (Fig. 1A and B). Computerised visual field (CVF) assessment was normal.

**Table 1:** Pre-operative laboratory results of case 1

**Parameter** (NV)	**Results**
Haematocrit (37–47%)	32%
Haemoglobin (11.5–16 g/dl)	11.2 g/dl
White blood cells (5000–10 000/mm^3^)	5680/mm^3^
Glycaemia (70–110 mg/dl)	88 mg/dl
Creatinine (0.5–1.2 ng/dl)	0.79 ng/dl
Sodium (135–145 mmol/l)	144 mmol/l
Potassium (3.5–5 mmol/l)	3.4 mmol/l
Total bilirrubin (0.1–1.4 ng/dl)	0.5 ng/dl
Alkaline phosphatase (31–100 UI/l)	87 UI/l
Aspartate aminotransferase (AST, 10–42 UI/l)	32 UI/l
Alanine aminotrasferase (ALT, 10–40 UI/l)	25 UI/l
Albumin (3.2–5 g/dl)	4.6 g/dl
Prolactin (5–25 ng/ml)	26 ng/ml
Luteinizing hormone (LH, 1–18 mU/ml)	1.3 mU/ml
Follicle-stimulating hormone (FSH, 4–13 mU/ml)	2.8 mU/ml
Estradiol (35–169 pg/ml)	<20 pg/ml
Growth hormone (<5 ng/ml)^a^	0.24 ng/ml
IGF1 (117–329 ng/ml)	100 ng/ml
Baseline cortisol (5–25 μg/dl)	9 μg/dl
Salivary cortisol 2300 h (0.7–5 nmol/l)	0.02 nmol/l
Free urinary cortisol 2400 h (<100 μg/2400 h)	20 μg/2400 h
Tyrotrophin (0.47–4.64 μU/ml)	0.6 μU/ml
Free thyroxine (0.7–1.8 ng/dl)	1.7 ng/dl
Anti TPO (<40 UI/ml)	0.9 UI/ml

NV, normal values; anti-TPO, anti-thyroperoxidase antibody; IGF1, insulin growth factor 1.

a No GH stimulation test was performed.

#### Treatment

Based on lesion size and image characteristics, the presumptive diagnosis was a macroadenoma, so glucocorticoid replacement therapy was started and the tumour later resected through a transsphenoidal approach with no complications. After surgery, amenorrhoea persisted and secondary hypothyroidism was detected. Oral contraceptives and thyroxine (50 μg daily) were prescribed.

#### Outcome and follow-up

**Figure 2:**
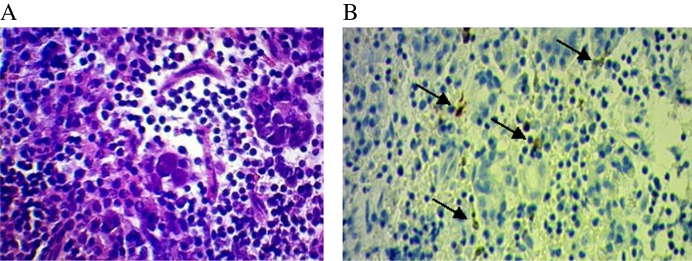
(A) Pituitary gland pathology report: dense inflammatory lymphoplasmacytic infiltrates. (B) IgG4-positive immunostaining in more than ten plasma cells per high power field (arrows, 40×).

Three months later a new MRI of the pituitary gland showed a persistent suprasellar tumour (Fig. 1C and D) in spite of lack of symptoms. Histopathology revealed adenohypophyseal tissue with dense inflammatory lymphoplasmacytic infiltrates (Fig. 2A). IgG4-positive immunostaining was observed in more than ten plasma cells per high power field (40×), confirming IgG4-related hypophysitis diagnosis (Fig. 2B). The new MRI findings and histologically confirmed diagnosis prompted a change in medication from hydrocortisone 15 mg daily to high-dose meprednisone; however, the patient did not accept this treatment although she finally agreed to take meprednisone 8 mg daily. MRI of the chest, abdomen, and pelvis did not reveal any pathology.

An MRI repeated after four months showed significant reduction in the pituitary mass lesion, demonstrating a good response to the low-dose steroids (Fig. 1E and F). After 20 months of follow-up there was no evidence of relapse of the pituitary mass or other organ involvement (not shown). She continued to persist with oral contraceptives and levothyroxine requirements.

### Case 2

#### Case presentation

A 37-year-old woman came for consultation because of severe headache, secondary amenorrhoea, polydipsia, and polyuria (urine output 10 l/day), weight loss and fatigue. Her past medical history noted a menarche at 12 years of age, no pregnancies and gluten-sensitive enteropathy that had improved shortly after starting a gluten-free diet. She was normotensive, and she had no abnormality detected on physical examination.

#### Investigation

**Figure 3:**
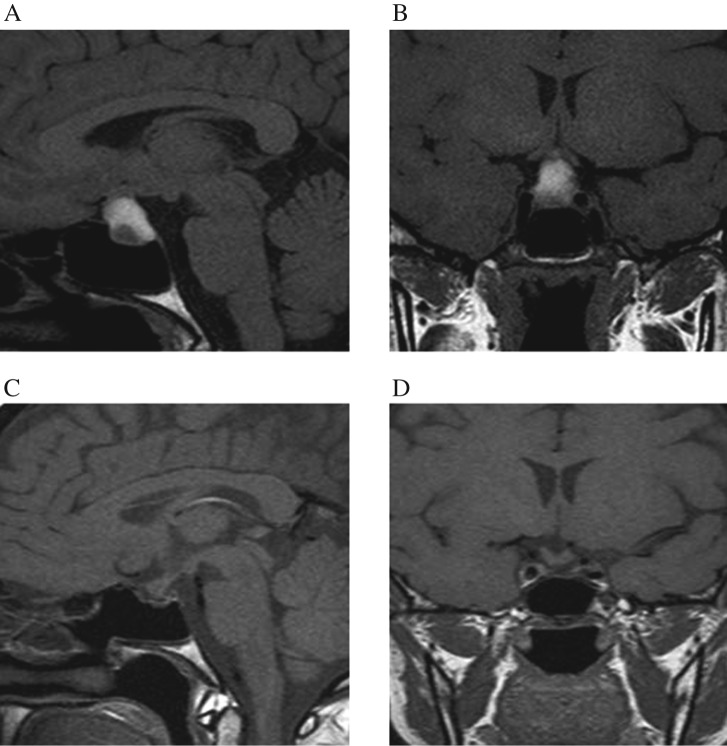
(A and B) Preoperative nonenhanced MRI: an extensive intrasellar lesion expanding into suprasellar region was found, with signs of bleeding, compression and optic chiasm displacement. (C and D) Postoperative nonenhanced MRI under meprednisone treatment showed significant reduction in sellar mass size.

The brain MRI showed a pituitary lesion extending into the suprasellar region with signs of intratumoural bleeding and compression of the optic chiasm (Fig. 3A and B). Baseline laboratory tests indicated panhypopituitarism and mild hyperprolactinaemia, interpreted as disconnection hyperprolactinaemia (Table 2). CVF showed left superior temporal quadrantinopia.

**Table 2:** Pre-operative laboratory results of case 2

**Parameter** (NV)	**Results**
Haematocrit (37–47%)	40%
Haemoglobin (11.5–16 g/dl)	13.6 g/dl
White cell blood count (5000–10 000/mm^3^)	4620/mm^3^
Glycaemia (70–110 mg/dl)	82 mg/dl
Creatinine (0.5–1.2 ng/dl)	0.87 ng/dl
Sodium (135–145 mmol/l)^a^	140 mmol/l
Potassium (3.5–5 mmol/l)	3.4 mmol/l
Total bilirrubin (0.1–1.4 ng/dl)	0.7 ng/dl
Alkaline phosphatase (31–100 UI/l)	49 UI/l
Aspartate aminotransferase (10–42 UI/l)	14 UI/l
Alanine aminotrasferase (10–40 UI/l)	21 UI/l
Albumin (3.2–5 g/dl)	4.1 g/dl
Luteinizing hormone (1–18 mU/ml)	0.19 mU/ml
Follicle-stimulating hormone (4–13 mU/ml)	1.26 mU/ml
Estradiol (35–169 pg/ml)	<25 pg/ml
Thyrotrophin (0.47–4.64 μU/ml)	<0.01 μU/ml
Free thyroxine (0.7–1.8 ng/dl)	0.83 ng/dl
IGF1 (117–329 ng/ml)	194 ng/ml
Cortisol (5–25 μg/dl)	4.4 μg/dl
Adreno corticotropin (10–46 pg/ml)	<5
Prolactin (5–25 ng/ml)	125 ng/ml

NV, normal value; IGF1, insulin growth factor 1.

a Under desmopressin treatment.

#### Treatment

Replacement treatment was started with hydrocortisone 15 mg/day, oral desmopressin 0.05 mg every 8 h, as well as the oral contraceptive. The patient underwent transsphenoidal surgery (TSS) with the presumptive diagnosis of pituitary macrodenoma with intratumoural haemorrhage.

**Figure 4:**
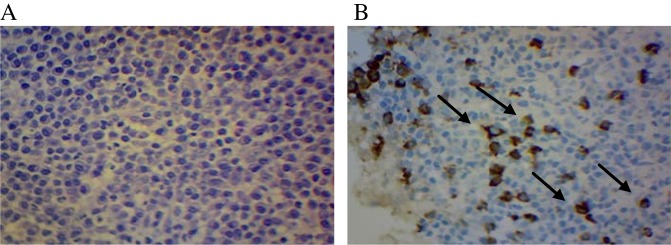
(A) Pituitary gland histopathology report: dense lymphoplasmacytic inflammatory infiltrates. (B) IgG4-positive immunostaining in more than ten plasma cells per high power field (arrows).

Histopathology results reported IgG4-related hypophysitis (Fig. 4A and B) and treatment was changed to meprednisone 16 mg daily. Computed tomography scans of the chest, abdomen, and pelvis were normal.

#### Outcome and follow-up

The patient's general health improved over subsequent follow-up. A repeat MRI performed 30 days after surgery showed a substantial reduction in sellar lesion size (Fig. 3C and D) and her CVF test improved. The patient continued clinical follow-up and treatment in her home town for eight months from diagnosis, and has not experienced a relapse of the hypophysitis nor any extrapituitary involvement. She remains with anterior hypopituitarism and diabetes insipidus.

## Discussion

IgG4-related disease is a fibro-inflammatory condition characterised by lesions with dense lymphoplasmacytic infiltrates, rich in IgG4-positive plasma cells, storiform fibrosis and often, though not always, high plasma IgG4 concentrations (6). Various disorders such as Riedel's thyroiditis, Mikulicz's disease, retroperitoneal fibrosis, tubulo-interstitial nephritis, and sclerosing cholangitis, among others, are now considered part of this disease spectrum (5).

IgG4-related hypophysitis was first described in 2004 based on clinical data (7); however, since 2007, the diagnosis normally requires histopathological confirmation (8). To date, 32 cases have been reported (5) (9).

In 2011, Leporati *et al*. established five criteria for IgG4 hypophysitis diagnosis: i) the presence of mononuclear cell infiltrates within the pituitary gland rich in plasma cells and lymphocytes, with more than ten IgG4-positive cells; ii) a sellar mass and/or thickened pituitary stalk; iii) biopsy-proven involvement of other organs, with IgG4-positive immunostaining; iv) increased IgG4 serum levels (>140 mg/dl); and v) reduction in sellar mass size and symptom improvement after corticosteroid treatment. The authors propose that IgG4-related hypophysitis diagnosis may be confirmed when any of the following criteria combinations are met: criterion i alone, criteria ii+iii or ii+iv+v (1).

Although the definitive diagnosis of hypophysitis is histopathological, imaging characteristics such as a symmetrical enlargement of the pituitary gland, rapid intense and uniform gadolinium enhancement, lack of erosion or depression of the sellar floor (2) and thickened pituitary stalk (9) should increase the diagnostic suspicion.

In our study, none of the patient presented characteristic pituitary changes detected by MRI for hypophysitis; instead, the images were heterogeneous and contrast enhancement was irregular. Moreover, in the first case there was considerable invasion of the sphenoid sinus and, in the second, signs of bleeding were present. Because these images did not initially suggest hypophysitis, both patients were submitted to TSS at which time definitive diagnosis of IgG4-related hypophysitis was established under the first Leporati's criterion, as also occurred in 11 of the 32 cases published to date (5) (9). Baseline IgG4 levels were not assayed since we did not initially suspect IgG4-related disease.

Of the 32 cases reported in the literature, 29 were men aged between 40 and 81 years, and 28 patients had other IgG4-related diseases, mostly pulmonary lesions, retroperitoneal fibrosis, autoimmune pancreatitis, lymphadenopathies, sialoadenitis, pachymeningitis, and kidney lesions (3). In contrast, both patients in our study were young women with no signs of clinical or radiological involvement of other organs at time of diagnosis.

With respect to clinical manifestations, both patients presented hypogonadotrophic hypogonadism and one had diabetes insipidus, in agreement with findings in the literature, where 25 of 32 cases presented varying degrees of hypopituitarism, 21 had diabetes insipidus and 16 patients presented both conditions (5) (9).

Concerning treatment, glucocorticoids are recommended as first-line therapy. More than half of the published cases received prednisolone (0.5–1 mg/kg per day), although some patients respond well to hydrocortisone replacement therapy (5). In our study, both patients presented good response to lower dose of meprednisone.

In conclusion, we report two cases of IgG4-related hypophysitis in young nulliparous women, the youngest to date, without typical radiological pituitary images, in whom the diagnosis was histologically confirmed. Notably, the disease was restricted to the pituitary and response to relatively low-dose glucocorticoids has been favourable.

## Patient consent

Written informed consent was obtained from the two patients.

## Author contribution statement

P Fainstein Day is the main Endocrinologist Physician who followed the patients and she reviewed and edited the manuscript. G A Sosa and S Bell conducted the literature review and manuscript construction. A Carrizo and P Ajler performed the surgeries. S B Christiansen was responsible for the histology-related aspect of both cases. M Pietrani was responsible for the imaging-related aspect of both cases. M Glerean, M Loto, and S Lovazzano contributed towards patient care as part of the Department of Endocrinology.
